# Supplementary material for: The interactome of intact mitochondria by cross-linking mass spectrometry provides evidence for coexisting respiratory supercomplexes
Source: Mol Cell Proteomics. 2017 Dec 8;17(2):216–32. doi: 10.1074/mcp.RA117.000470 (PMC5795388; doi:10.1074/mcp.RA117.000470)
Supplement: Supplemental Data [file supp_RA117.000470_133922_0_supp_23978_fzffwf.zip › spectra_annotation/mito_spectra_annotation/382-1-1-1-21-1.pdf]

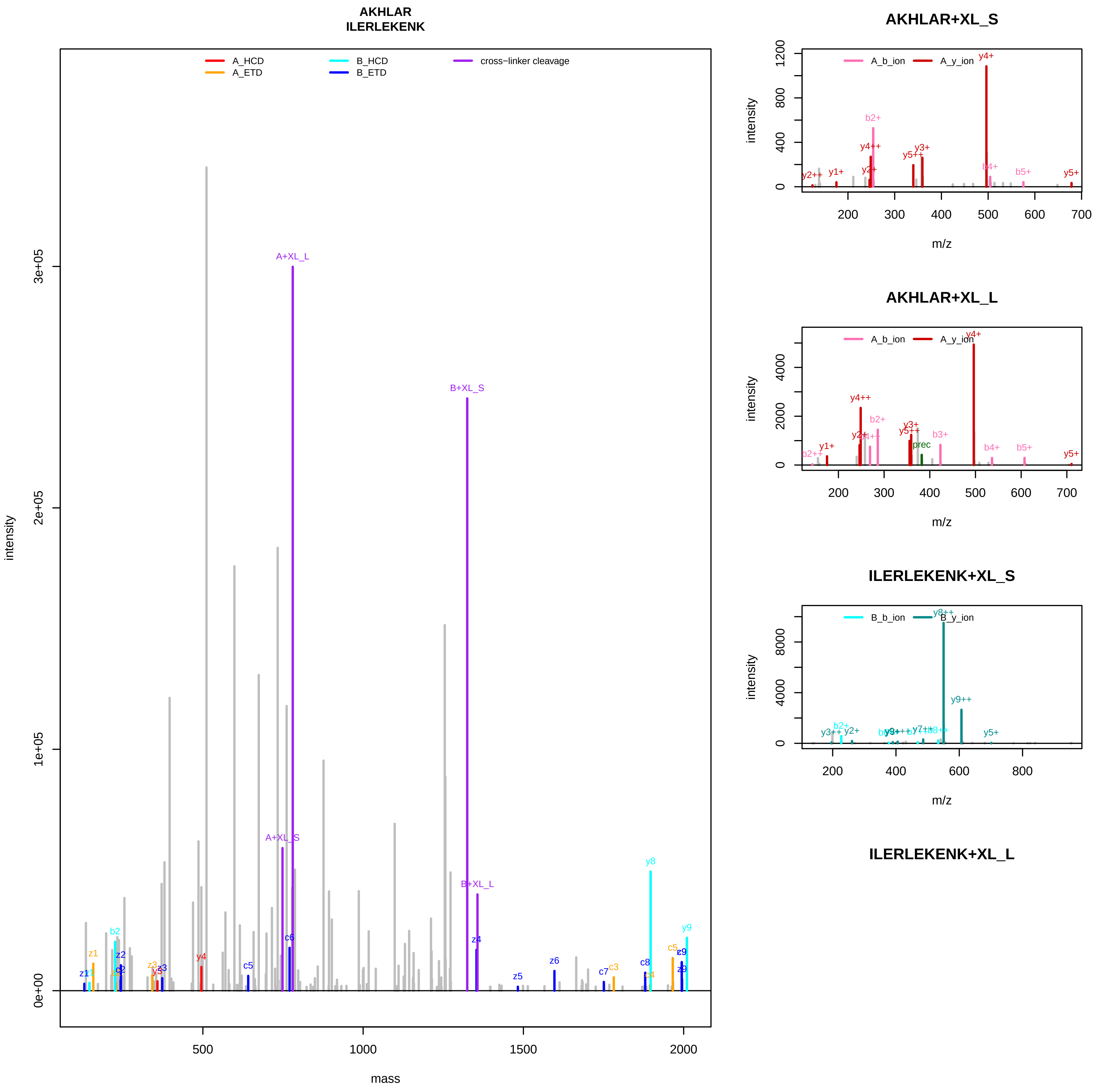

AKHLAR
ILERLEKENK
A_HCD
A_ETD
B_HCD
B_ETD
cross−linker cleavage
A+XL_L
B+XL_S
A+XL_S
B+XL_L
y8
y9
b2
c5
c6
c7
c8
c3
c4
z1
z2
z3
z4
z5
z6
z9
y4
y3
intensity
mass
0e+00
1e+05
2e+05
3e+05
500
1000
1500
2000
AKHLAR+XL_S
A_b_ion
A_y_ion
y4+
b2+
y4++
y3+
y5++
y2+
y1+
y2++
b4+
b5+
y5+
0
400
800
1200
200
300
400
500
600
700
m/z
AKHLAR+XL_L
A_b_ion
A_y_ion
y4+
y4++
b2+
y3+
y5++
b3+
prec
y1+
b2++
b4+
b5+
y5+
0
2000
4000
m/z
ILERLEKENK+XL_S
B_b_ion
B_y_ion
y8++
y9++
b2+
y2+
y3++
y7++
y5+
0
4000
8000
200
400
600
800
m/z
ILERLEKENK+XL_L
